# Uptake of Eudragit Retard L (Eudragit^®^ RL) Nanoparticles by Human THP-1 Cell Line and Its Effects on Hematology and Erythrocyte Damage in Rats

**DOI:** 10.3390/ma7031555

**Published:** 2014-02-28

**Authors:** Mosaad A. Abdel-Wahhab, Khaled G. Abdel-Wahhab, Fathia A. Mannaa, Nabila S. Hassan, Ramia Safar, Roudayna Diab, Bernard Foliguet, Luc Ferrari, Bertrand H. Rihn

**Affiliations:** 1 Food Toxicology & Contaminants Department, National Research Center, Dokki, Cairo 12311, Egypt; 2 Medical Physiology Department, National Research Center, Dokki, Cairo 12311, Egypt; E-Mails: kgm194@yahoo.com (K.G.A.-W.); fathia_98@yahoo.com (F.A.M.); 3 Pathology Department, National Research Center, Dokki, Cairo 12311, Egypt; E-Mail: nabilas.hassan@yahoo.com; 4 Faculty of Pharmacy, EA 3452 CITHEFOR (Cibles thérapeutiques, formulation et expertise préclinique du médicament), Lorraine University, Nancy Cedex 54001, France; E-Mails: ramia.safar@univ-lorraine.fr (R.S.); roudayna.diab@univ-lorraine.fr (R.D.); bernard.foliguet@univ-lorraine.fr (B.F.); luc.ferrari@univ-lorraine.fr (L.F.)

**Keywords:** Eudragit, nanoparticles, nanotoxicity, hematology, erythrocytes damage, oxidative stress

## Abstract

The aim of this study was to prepare Eudragit Retard L (Eudragit RL) nanoparticles (ENPs) and to determine their properties, their uptake by the human THP-1 cell line *in vitro* and their effect on the hematological parameters and erythrocyte damage in rats. ENPs showed an average size of 329.0 ± 18.5 nm, a positive zeta potential value of +57.5 ± 5.47 mV and nearly spherical shape with a smooth surface. THP-1 cell lines could phagocyte ENPs after 2 h of incubation. In the *in vivo* study, male Sprague-Dawley rats were exposed orally or intraperitoneally (IP) with a single dose of ENP (50 mg/kg body weight). Blood samples were collected after 4 h, 48 h, one week and three weeks for hematological and erythrocytes analysis. ENPs induced significant hematological disturbances in platelets, red blood cell (RBC) total and differential counts of white blood cells (WBCs) after 4 h, 48 h and one week. ENP increased met-Hb and Co-Hb derivatives and decreased met-Hb reductase activity. These parameters were comparable to the control after three weeks when administrated orally. It could be concluded that the route of administration has a major effect on the induction of hematological disturbances and should be considered when ENPs are applied for drug delivery systems.

## 1. Introduction

The quantity of nanoparticles (NPs) produced annually is rapidly increasing [1], fueled by growing markets for products that incorporate these materials. This trend will likely lead to the appearance of human-made NPs in air, water, soils and organisms [2]. The unique physicochemical properties of NPs that have given rise to applications in many fields, including drug delivery [3], cancer therapy [4], biosensors [5], food additives and cosmetics [6]. Possible toxicological risks associated with NP exposure may arise during material fabrication, handling, usage and waste disposal [7,8]. Consequently, the risk of toxicity to humans or the environment will increase [9,10].

Because of their small size, NPs have many physicochemical properties that differ from those of their bulk forms. Recent studies have indicated that the physicochemical characteristics of NPs mainly, the size, shape, surface area, solubility, chemical composition and dispersion factor, play critical roles in determining their biological responses [9,11,12]. For example, NPs of a smaller size can enter the mitochondria of cells through various pathways, subsequently inducing oxidative stress and cell death via apoptosis [13] or autolysis [14].

Polymeric NPs have been used as a preferred nanoscale drug delivery vehicle, especially for their excellent endocytosis efficiency, passive tumor-targeting, high encapsulation efficiency and delivery of a wide range of therapeutic agents [15]. Eudragit is a model polymeric NP, and it is a copolymer that has been widely used to improve the solubility of poorly water soluble drugs [16]. Eudragit RS (ERS), a non-biodegradable, positively-charged copolymer, has been licensed for clinical use by the major health authorities of Europe, Japan and the USA [17] as an efficient nano-drug delivery system (NDDS). ERS NPs prepared by nanoprecipitation (NP) or by double emulsion (DE) techniques containing ibuprofen and cyclosporin [18], indomethacin [19], melatonin [20], DNA plasmid [21] or low-molecular weight heparin [22] (LMWH) have been obtained and been suggested for therapeutic usage. On the other hand, Eudragit Retard L (Eudragit RL) polymer NPs have been investigated as carrier systems for the ophthalmic release of non-steroidal anti-inflammatory drugs, such as ibuprofen or flurbiprofen [18,23]. Moreover, Eudragit L 100-55 (EL 100-55 methacrylic acid-ethyl acrylate copolymer type A, 1:1) has been commonly used for the preparation of enteric solid dosage forms as a good coating and skeleton material [24,25]. The aim of the current study was to develop Eudragit RL NPs as a delivery for several drugs and to evaluate their properties, uptake by the human THP-1 cell line *in vitro* and effect on the hematological parameters *in vivo* using an animal model. In a previous study, Eudragit RL nanoparticles (ENPs) were used to encapsulate heparin for oral administration and were tested *in vivo* on rabbit [22]. Subsequently, it was necessary to assess their short-term toxicity and to evaluate the intraperitoneal route, because it is widely used for chemotherapy in patients with gastrointestinal and gynecological cancers [26], compared to the oral route.

## 2. Results

**Figure 1.**
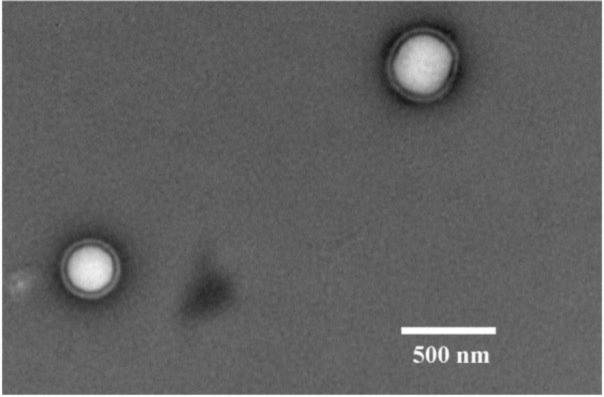
Transmission electron microscopy (TEM) image of the prepared Eudragit Retard L (Eudragit RL) nanoparticles (ENPs).

**Figure 2.**
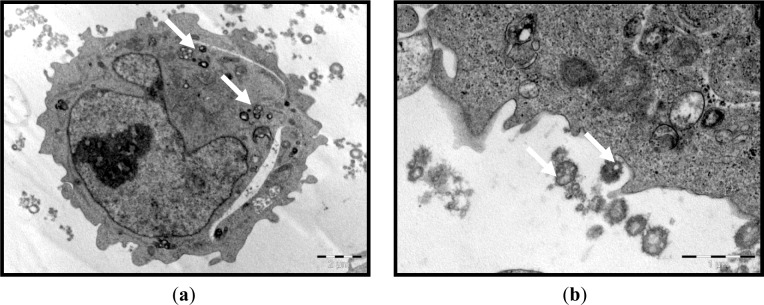

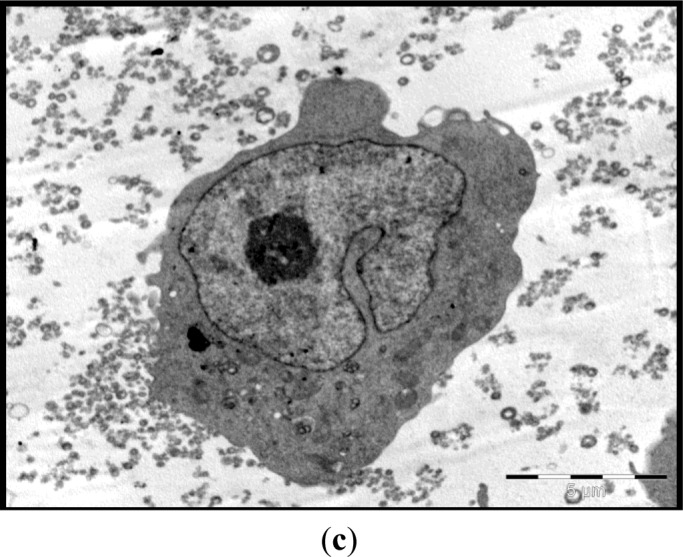
TEM of ENP uptake by the human THP-1 cell line. Monocytes were exposed to 200 μg/mL for 2 h; the arrows indicate (**a**) internal; (**b**) external nanoparticles; and (**c**) that no changes were observed in the cell structure after 24 h.

We first established that the characteristics of the prepared ENPs were an average size of 329.0 ± 18.5 nm and positive zeta potential values of 57.5 ± 5.47 mV. The prepared ENPs did not show any changes in their size in neutral or acidic conditions, indicating that no aggregation had occurred, except when serum was added to the media. The transmission electron microscope (TEM) images showed that the ENPs were nearly spherical in shape with a smooth surface (Figure 1). Moreover, in the *in vitro* study, the TEM observations revealed that the uptake of ENPs by THP-1 cell lines occurred after 2 h of incubation with a concentration of 200 μg ENP/mL (Figure 2a,b). The ENPs were observed in the cells after 24 h without inducing any changes in the cell structure (Figure 2c).

The results presented in Table 1 illustrated the effect of the route (oral and intraperitoneally; i.p) of administration on animals’ total and differential count of white blood cells (WBCs), as well as the platelet count at different time intervals as compared to the control group. These results revealed that after 4 h of ENP treatment, the platelet count recorded a significant decrease regarding IP injection and still close to normal in the case of oral administration. The total WBC count was not affected, while its differential count was changed, as the granulocyte percentage was significantly elevated after IP injection. This elevation could be on the expanse of the lymphocyte percentage, which was insignificantly decreased. The percentage of monocytes did not significantly change by comparison of the routes of treatment to each other, as well as with the control group.

After 48 h of IP injection of ENPs, the platelet count significantly increased compared to its count after 4 h and became close to that of the normal group or its corresponding value of those treated orally. The total WBC count of both the IP and oral groups was significantly increased. Their differential count was changed, as the granulocyte percentage was significantly elevated in both the IP and oral cases. This elevation could be on the expanse of the lymphocyte percentage, which decreased insignificantly. On the other hand, the percentage of monocytes did not significantly change, either by comparing the routes of treatment to each other or with the control group.

One and three weeks after either oral or IP treatment with ENPs, the obtained data showed that the platelet count was slightly elevated over the control, while the total count of WBCs decreased gradually towards the control range. However, the decline was slower regarding IP injection. In addition, the WBC differential count showed a reduction in the percentage of granulocytes accompanied with an elevation in lymphocyte percentage; however, monocytes were again more or less unaffected significantly.

The results of blood values presented in Table 2 revealed that both oral administration and IP injection with ENPs showed a time-dependent gradual change, but not an administration-route-dependent change. The data illustrated that animals either orally administrated or IP injected with ENPs showed a marked decrease in red blood cell (RBC) count and HCt percentage after four or 48 h and returned to the control range by the other intervals. No sharp difference was observed between both routes at the matched time intervals. Similarly, Hb, mean corpuscular hemoglobin (MCH) and mean corpuscular hemoglobin concentration (MCHC) levels recorded a time-dependent gradual decrease at all tested intervals, although this decrease was slightly pronounced after 4 h and significant after seven days for Hb concentration only. Instead, no significant differences were found in MCHC between the control and the ENP-treated groups. In contrast, the mean corpuscular volume (MCV) level showed a time-transit elevation in all groups, either orally administrated or IP injected with ENPs. This increase was markedly pronounced after one week for both treatments with ENPs. At the end of the third week, all blood values were carried up or down, more or less, towards the control values.

The data of both functional (O_2_-Hb) and nonfunctional (Met-Hb, S-Hb and Co-Hb) hemoglobin derivatives of different ligands are illustrated in Table 3. The current results showed that after IP or oral administration of ENPs, the functional derivative (oxy-Hb) or the nonfunctional one (S-Hb) were not affected over all the tested time intervals, while the nonfunctional fraction Co-Hb was found to be elevated significantly after 4 h, 48 h and one week, whatever the exposure route, and to fall down near the control range. Furthermore, the nonfunctional met-Hb derivative showed a time/route gradual elevation, which was significant after three weeks of IP injection (Table 3).

**Figure 3.**
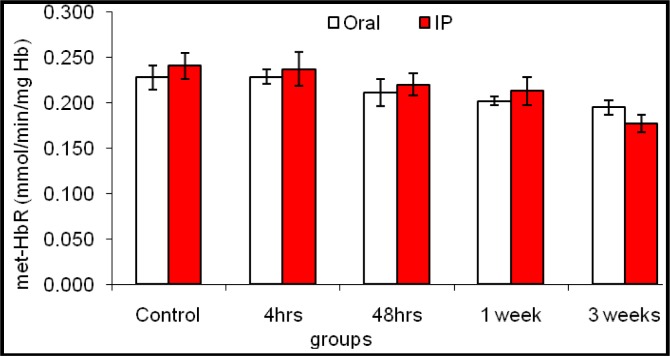
The effect of oral administration and intraperitoneal injection of ENPs on the erythrocyte methemoglobin reductase (metHbR) activity of rats at different time intervals.

In contrast, the obtained data illustrated that the activity of met-HbR was comparable to that of the control after four and 48 h, while a significant decrease was pronounced after the first and third weeks in both routes of treatments (Figure 3).

## 3. Discussion

NPs (nanoparticles) can be transported around the body and be taken up by organ tissues and cell cultures, resulting in increased oxidative stress, inflammatory cytokine production or cell death. Unlike larger particles, NPs may be taken up by cell mitochondria, and cell studies demonstrated the potency of NPs to induce: (i) DNA mutation; (ii) major structural damage to mitochondria; and even (iii) cell death [27,28]. From this point of view, NPs used for medical applications should be evaluated *in vivo*, as well as *in vitro*. Polymeric NPs are biocompatible, surface-modifiable and capable of sustained drug release. They show potential applications in the treatment of various pulmonary conditions, such as asthma, chronic obstructive pulmonary disease (COPD), tuberculosis (TB) and lung cancer, as well as in diabetes [29–33].

In the current study, the average size of the developed Eudragit RL NP (ENP) was 329.0 ± 18.5 nm and the zeta potential was +57.5 ± 5.7 mV. From a pharmaceutical point of view, what is most important is the increase in saturation solubility and the adhesiveness to surfaces/membranes. According to the Kelvin equation, there is an increase in saturation solubility (Cs) below ~1 μm [34], this being more pronounced for poorly soluble than for soluble drugs [35]. The smaller the material is, the more adhesive it is. It is well known that the particle sizes of Eudragit RL and others polymers is affected by the preparation method. The double emulsion method was applied in the current study for the preparation of ENPs, which cannot produce a smaller size of NP (*i.e*., 30 nm). However, Eidi *et al*. [14] used the nanoprecipitation method to produce a smaller size of Eudragit RS NPs, which have reached 55 nm. On the other hand, particle size can be affected by the different pH and ionic strength used during administration. However, in the current study, the size of the nanoparticles was investigated in neutral (pH 7) and acidic (pH 3) solutions. The results showed that the preparation of ENPs with a size of 330 nm did not show any aggregation in the neutral or acidic conditions, except when the serum was added to the media.

Both pharmaceutically positive effects justify the definition of NPs with a size below 100 nm, and this size limit should be considered when defining a classification system. Although 100 nm is the second important size limit, the larger particles can enter the cell only by phagocytosis. Consequently, these particles can be taken up only by monocytes, a limited cell population in the body. In addition, many of them are difficult to access, e.g., to be taken up by liver and spleen monocytes, the particles need to enter the blood stream. Therefore, the developed ENPs possess a lower toxicity risk. However, particles below 100 nm can be internalized by certain cells by endocytosis or clathrin-dependent mechanism. Thus, these particles have a higher toxicity risk, and the 100 nm limit also has to be considered in the classification system [36].

Zeta potentials provide quantitative information on the stability of the particles. In the current study, ENPs had an absolute value of zeta potential higher than 30 mV (+ 57.5 ± 50.7 mV). It is well documented that the particles are more likely to remain dispersed if the absolute value of zeta potential is higher than 30 mV [37]. The current results demonstrated that ENPs possessed a uniform shape along with relatively favorable dispersibility. The uptake was performed within the concentration range and time period, when no damage to cell viability confirmed the low cytotoxicity of the assayed polymeric ENPs to THP-1 cells (data not shown). This could lead to a true understanding of the relationship between particle size, as well as surface charge and cellular uptake. The THP-1 cells succeeded in inducing the uptake of ENPs (Figure 2). However, no difference was observed between the cells treated with a low or high dose of ENPs. On this concern, He *et al*. [38] reported that positively charged NPs showed a higher phagocytic uptake compared to negatively charged nanoparticles. However, Zauner *et al*. [39] suggested that the penetration of NPs into the cells might be attributed to the impairment of the cell membrane structure. It is well documented that the toxic effect of NPs is highly dependent on the organs and, more specifically, the type of cell encountered. This is due to the variation in cell physiology (e.g., epithelial or lymphoid), proliferation state (tumor or resting cells), membrane characteristics and phagocyte characteristics among different cell types [40]. The choice of the THP-1 cell line in the current study was based on the fact that these monocytes are the first line of innate immunity and, therefore, represent the first “barrier” or “target” of nanoparticulate delivery systems. On the other hand, the major objective of the current study was to investigate the effect of NPs on the hematological parameters, to assess the effect of the physical properties of ENPs on their toxicity and the possibility of NPs to penetrate the THP-1, as an example of blood cells.

The effect of ENPs on vascular connective tissue and hematological parameters was evaluated *in vivo*. NPs can translocate to both circulatory and lymphatic systems and, ultimately, to other body tissues and organs. However, translocation differs according to the route of exposure to nanoparticles [41]. In this study, both total and differential counts of WBCs were significantly changed at most tested time intervals. A significant elevation in both WBC count and granulocyte percentage was noticed after 48 h of oral or IP exposures. The elevation of the later was probably on the detriment of lymphocyte percentage, which decreased non-significantly. By the third week, the WBC of the oral group declined close to control values, while that of the IP group still elevated significantly compared to the PO exposed group.

Several reports suggested that high concentrations of NP can enter into the lymphatic system, inducing enlargement and inflammation in lymph nodes, which helps to increase the number of WBCs. However, after a given period, the activity of these glands decreased, and lymph node atrophy has been noted [42]. This finding is also supported by Choi *et al*. [43]. Some nanoparticles, depending on their composition and size, can produce irreversible damage to cells by oxidative stress or/and organelle injury [41]. ENPs can be taken up by various cell types, resulting in increased oxidative stress and inflammatory cytokine production. Regarding this concern, do Carmo *et al.* [44] reported that these effects trigger a chronic inflammatory condition that is associated with a high white blood cell count. Moreover, neutrophils, through NCF-1 and NADPH oxidase activation, increase phagocytosis and the formation of reactive oxygen compounds, as well as their own stimulation and migration to inflammatory sites.

Platelet count was decreased significantly after 4 h of ENP IP injection and returned to the control range by the other time intervals. As is known, NP charge plays an essential role in their uptake by platelets and their influence on blood clot formation [45]. Negatively charged NPs significantly inhibit thrombus formation, while positively charged NPs enhance platelet aggregation [46]. The interaction between platelets and positively charged particles seems to be due to the net negative charge that platelets carry on their surface [47]. In addition, the positively charged NPs interact with negatively charged platelets and reduce their surface charge, making them more prone to aggregation. This could be the possible reason for the thrombocytopenia occurring after 4 h of ENP injection. Until now, it was thought that blood clots can be formed due to three main causes: when the blood flow is obstructed or slowed down, when the vascular endothelial cells are damaged or due to the blood biochemistry. Moreover, due to the large ENPs used in the current study, they failed to produce a thrombotic effect, suggesting that these NPs are insufficient for causing peripheral thrombosis [48].

In the current study, animals exposed to either oral or the IP treatment showed a marked decrease in RBC count and HCt percentage after four and 48 h, which returned to the control range by the other intervals with no valuable difference between both routes at the same time interval. Similarly, Hb, MCH and MCHC levels recorded a time-dependent gradual decreases at all tested times, although this decrease was slightly pronounced after 4 h (reflecting hypochromia) after seven days for Hb. In contrast, the MCV level showed a time-transit elevation (resulting in macrocytosis), especially after one week for both routes of treatment. These results suggested that the exposure route of ENPs is important and should be considered in the application of ENPs. Generally, the disturbances in erythrocytes and hemoglobin reported herein suggested that both bone marrow and erythrocytes may undergo a transient oxidative stress, as this damage was overcome by the third week. A high concentration of cerium oxide NPs reduced the number of blood cells due to: (i) inhibition of cell activity; (ii) antimitotic properties; (iii) stimulation of oxidative stress in cells; (iv) reduction of cellular antioxidants; and (v) activation of immune cell processes. Those effects may result in erythrocytopenia (low RBCs) and hypochromia via either a reduction of bone marrow activity, reducing, consequently, the RBC production rate, or the impairment of RBC membranes by making them more fragile and increasing their hemolytic rate. On this concern, Machiedo *et al*. [49] demonstrated that free radicals that were produced by NPs can be the main cause for the destruction of red blood cells by lipid peroxidation. Susan *et al*. [50] showed that their distribution in tissues and their effect change with respect to the diameter of the nanoparticles; if diameters are smaller, the influence of NPs on molecular mechanisms increases [45,51].

Both functional anemia (an elevation in nonfunctional Hb derivatives) and physiological anemia may evidence a weakness (erythrocytopenia, hypochromic-macrocytosis) in the oxygen supply of tissues (tissue hypoxia) at the intervals between the first and third week. Moreover, the current results showed that IP or PO administration did not affect either the functional derivative (O_2_-Hb) or the nonfunctional derivative (S-Hb), while the nonfunctional derivative, Co-Hb, was found to be elevated significantly 4 h, 48 h and one week after the treatment through both routes, then falling down near the control range. Unfortunately, the nonfunctional met-Hb derivative recorded a time/route gradual elevation, which was significant after three weeks of IP injection with ENPs. Moreover, the data illustrated in Figure 3 revealed that the activity of met-HbR was comparable to the control after four and 48 h, while a significant inhibition was pronounced after the first and third weeks in both routes of treatment, as compared to the control values. It is well documented that in most cells, mitochondria are a major source of ROS [46]. Despite their lack of mitochondria, ROS are continuously produced in the erythrocytes, due to the high O_2_ tension in arterial blood and their abundant heme iron content, inducing Fenton chemistry reactions [52]. The source of ROS in erythrocytes is the oxygen carrier protein hemoglobin, oxyhemoglobin (Oxy-Hb), which undergoes autoxidation to produce O_2_^•−,^ which yields hydrogen peroxide [53]. Exposure of Hb to H_2_O_2_ leads to oxidative modifications that have been proposed as selective signals for proteolysis in erythrocytes [54]. The occasional reduction of O_2_ to O_2_^•−^ is accompanied by the oxidation of the functional ferrous-oxyhemoglobin (O_2_-Hb) to the nonfunctional ferric-methemoglobin (Met-Hb), a “rust” brown-colored protein that cannot bind and transport O_2_ [46]. This process is reversed by the presence of an efficient met-Hb reductase system in erythrocytes, comprising various enzymatic and non-enzymatic mechanisms. Moreover, the accumulation of a substantial amount of met-Hb in erythrocytes causes methemoglobinemia, clinically manifested by dyspnea and cyanosis [55]. Eventually, oxidative stress can lead to organelle injury and the formation of Hienz bodies, a form of methemoglobinemia, that precipitate on the inner side of the RBC membrane, triggering cell hemolysis [48]. Additionally, met-Hb can then react with the peroxides formed during the autoxidation process itself or elsewhere, resulting in hematological disorders [56–58].

Met-Hb is formed when the ferrous porphyrin complex of Hb is oxidized into the ferric form [59]. *In vivo*, Met-Hb is predominately reduced mainly by the NADH cytochrome b5-Met-Hb reductase system and to a lesser extent by the NADPH-dependent Met-Hb reductase enzyme [53]. It was suggested that intracellular NADPH concentration may be important in preventing Met-Hb generation. Loss of NADPH and glutathione (GSH) is thought to account for the enhanced rates of Met-Hb generation, as well as lipid peroxidation [52,60]. The free radicals may also induce configuration changes in the Hb molecule and make it susceptible to binding unfavorable ligands other than oxygen, such as carbon monoxide (CO) and sulfur (S). In addition to its inhibition of Met-Hb reductase activity, oxidative stress induced by NPs may also reduce GST, CAT and GPx, as well as GSH level in erythrocytes. Damodara and Venkaiah [55] suggested that the oxidative stress is normally challenged in erythrocytes by cellular defenses, including these antioxidant agents, which are important for dealing with the endogenous H_2_O_2_ produced by Hb autoxidation. The increased oxidative stress in erythrocytes could lead to the exhaustion of the antioxidant battery, which becomes insufficient to counteract the excessive production of ROS [61]. ROS was previously shown to inhibit RNA synthesis in liver and, therefore, affecting enzyme content [62]. In addition, metal interactions with hydrogen peroxide have been frequently implicated in catalyzing free-radical-mediated damage. Redox-active transition metals, such as iron, containing unpaired electrons in their d-orbitals are able to generate reactive species by Fenton Haber Weiss chemistry. In particular, free ferrous iron has been suggested to catalyze tissue damage by reacting with hydrogen peroxide to generate the highly reactive hydroxyl radical [63]. Taken together, these results revealed that ENPs could induce slight oxidative stress. However, the body succeeded in counteracting it after three weeks of treatment. The current study indicated that all blood cells of rats treated with ENPs showed slight changes in their physiological parameters. Consequently, we hope these studies can introduce a way to prevent and are aware of the toxicity effects of NPs in human health.

## 4. Experimental Section

### 4.1. Chemicals

Eudragit^®^ RL PO (M_W_ = 150,000 Da (CAS number: 33434-24-1)), an acrylic polycationic copolymer of acrylic and methacrylic acid esters with a proportion of quaternary ammonium groups (0.5%–0.8%), was a generous gift from Evonik polymers (Darmstadt, Germany). Pluronic F68 (CAS number: 11104-97-5) was used as a surfactant and was obtained from Sigma Aldrich (Saint-Quentin Fallavier, France). RPMI medium was purchased from GIBCO (Invitrogen, Cergy Pontoise, France).

### 4.2. Preparation of Eudragit^®^ RL Nanoparticles

Eudragit^®^ RL NPs (ENP) were prepared using the double emulsion/solvent evaporation technique, as described by Bodmeier *et al*. [64]. Briefly, 500 mg of Eudragit^®^ RL were dissolved in 5 mL of an organic solution, dichloromethane (DCM, Laurylab, Saint Fons, France). One half milliliter of a surfactant aqueous solution at 0.1% w/v pluronic^®^ F68 was emulsified into this organic phase by sonication (40 W for 1 min) using an ultrasonic homogenizer (Vibracell 75022, Bioblock, Illkirch, France) for the preparation of the primary w/o emulsion. This primary w/o emulsion was then dispersed by sonication (80 W for 30 s) into 20 mL of an aqueous solution of pluronic^®^ F68 (0.1%, w/v), thus producing a secondary w/o/w emulsion. The resulting ENPs were obtained by the evaporation of the organic phase. The formed nanoparticles were separated by ultracentrifugation (Beckman, Miami, FL, USA) at 55,000 rpm for 30 min. The obtained nanoparticles were then re-suspended in an appropriate medium for further investigations. For the *in vitro* and *in vivo* studies, the weight of ENPs has been calculated based on the polymer concentration in the prepared NP suspension considering the density of the Eudragit polymer: 1 μg Eudragit polymer corresponds to 8.23 × 10^7^ of ENPs.

### 4.3. Characterization of ENPs

#### 4.3.1. Particle Size and Zeta Potential Analysis

Particle sizes were determined by photon correlation spectroscopy (PCS) using a Zetasizer™ 3000E (Malvern Instruments Worcestershire, UK). To avoid multi-scattering events, each sample was diluted with double distilled water until the appropriate concentration of particles; the concentration and dilution were kept constant for all samples. The particle size (z-average) and size distribution of equivalent hydrodynamic spheres were calculated using the Malvern associated software and an exponential sampling method. The size of the prepared ENPs was tested at neutral (pH 7) and acidic (pH 3) conditions, and each measurement was performed in triplicate. Zeta potential measurement was based on nanoparticle electrophoretic mobility and calculated from Smoluchowski’s equation [65]. All measurements were performed in triplicate at 25 °C.

#### 4.3.2. Morphological Determination

The morphological determination of ENPs was analyzed by transmission electron microscopy (Stereoscan 240 S/N Léo, Rueil-Malmaison, France). Briefly, a drop of the fresh ENP sample was placed onto a carbon-coated copper grid, forming a thin liquid film, which was negatively stained by the addition of a drop of uranyl acetate. The excess of the staining solution was removed with filter paper and then air-dried before the observation. Image acquisition was done with an Orius 1000 CCD camera (GATAN, Pleasanton, CA, USA).

### 4.4. In Vitro Study

#### 4.4.1. Cell Culture

The human THP-1 cell line was obtained from American Type Culture Collection (ATCC, Manassas, VA, USA). Cells were grown in RPMI 1640 medium (GIBCO, Invitrogen, Cergy Pontoise, France) supplemented with 10% of heat-inactivated fetal bovine serum (Eurobio, Les Ullis, France), as well as 100 U/mL of penicillin, 100 μg/mL of streptomycin and 0.25 μg/mL of amphotericin B (all from Sigma, Saint Quentin Fallavier, France). Cells were incubated at 37 °C under 5% CO_2_ atmosphere and split every 3 days.

#### 4.4.2. Cellular Uptake Visualization by Transmission Electronic Microscopy (TEM)

Briefly, THP-1 cells were seeded in 6-well plates at a density of 3 × 10^5^ cell/well and treated on the following day with 200 μg/mL of ENPs for 2 h or 24 h. Then, they were immediately fixed with ice-cold 2.5% glutaraldehyde for 2 h. Cells were then post-fixed in 1% OsO_4_ for 1 h at room temperature, progressively dehydrated by increasing concentrations of ethanol and, finally, treated with propylene oxide and included in resin. Semi-fine (2.0 μm) and ultra-fine (90 nm) sections were prepared with an ultra-microtome (Reichert-Yung) and examined with the electron microscope, Philips CM 12, operated at 80 kV.

### 4.5. In Vivo Study

#### 4.5.1. Experimental Animals

Three-month-old male Sprague-Dawley rats (150–180 g, purchased from Animal House Colony, Giza, Egypt) were maintained on standard lab diet (protein: 160.4; fat: 36.3; fiber: 41 g/kg; and metabolizable energy: 12.08 MJ) purchased from Meladco Feed Co. (Aubor City, Cairo, Egypt). Animals were housed in filter-top polycarbonate cages in a room free from any source of chemical contamination, artificially illuminated and thermally controlled, at the Animal House Lab., National Research Center, Dokki, Cairo, Egypt. All animals received humane care in compliance with the guidelines of the Animal Care and Use Committee of the National Research Center, Dokki, Cairo, Egypt.

After an acclimatization period of one week, the animals were divided into two main groups (50 rats/group), and each group was divided into 5 subgroups (10 rats/subgroup). Ten rats from the first main group were injected intraperitoneally (i.p.) with saline solution and used as the control group, and the remaining rats were injected intraperitoneally (i.p.) with a single dose of ENPs (50 mg/kg body weight; b.w) suspended in saline. Ten rats from the second main group received a single oral dose of saline solution and were used as the control group, while the remaining rats received a single oral dose of ENPs (50 mg/kg b.w) using a stomach tube. Blood samples were collected from the two control groups after 4 h of saline treatment and after 4 h, 48 h, 1 wk and 3 wk of exposure from all ENP treated animals (10 rats/each time period) under diethyl ether anesthesia in two clean heparinized tubes; the first was used immediately for performing complete blood count (CBC), and the second part was used in the preparation of hemoglobin solution for the determination of hemoglobin derivatives of different ligands, as well as methemoglobin reductase (Met-HbR) assessment.

A fully automatic blood cell counter with a double capillary instrument (Model PCE-210N, ERMA INC, Tokyo, Japan) was used for measuring complete blood count [red blood cell (RBC) count, total hemoglobin (Hb) concentration, hematocrite (Hct) percentage, platelet (PLT) count and white blood cells (WBCs) total and deferential counts (lymphocytes, monocytes and granulocytes)] as well as blood indexes [mean corpuscular volume (MCV), mean corpuscular hemoglobin (MCH) and mean corpuscular hemoglobin concentration (MCHC)].

#### 4.5.2. Hemolysate Preparation and Determination of Hb Derivatives of Different Ligands

Hemolysate was prepared according to the method described by Silva *et al*. [66]. Briefly, whole blood was centrifuged at 3000 rpm for 15 min, and the buffy coat was removed. The packed red cells were washed three times with physiological saline, and the washed cells were lysed by suspending in hypotonic phosphate buffer and centrifuged at 7000 rpm for 30 min. The resulting pellet is the erythrocyte membrane, and the supernatant represents the hemolysate. The hemolysate obtained was further used for the assessment of both functional and nonfunctional hemoglobin derivatives of different ligands.

#### 4.5.3. Hemoglobin Derivatives of Different Ligands Determination

Hemoglobin derivatives of different ligands were determined as a percent of total hemoglobin. The methemoglobin (Met-Hb) level was determined in the blood sample using the method described by Evelyn and Malloy [67]. Oxyhemoglobin (O_2_-Hb), sulfhemoglobin (S-Hb) and carboxyhemoglobin (Co-Hb) levels in the blood sample were determined spectrophotometrically according to the method described by Van Kampen and Julstra [61].

#### 4.5.4. Met-Hemoglobin (Met-Hb) Reductase Activity

Met-hemoglobin reductase (Met-HbR) activity was assayed in the washed packed erythrocytes according to the method of Hegesh *et al*. [62].

### 4.6. Statistical Analysis

All data were subjected to statistical analysis using the general linear model procedure of the statistical analysis system. Comparisons between means were carried out using a multiple one-way (Duncan) ANOVA test at the level of *p* ≤ 0.05 [63] using the statistical analysis system (SAS) program software; copyright© 1998 by SAS Institute Inc., Cary, NC, USA.

## 5. Conclusions

It could be concluded from the current study that the ENPs are internalized by the THP-1 cell line. ENPs induced moderate hematological disturbances, represented in platelet, total and differential counts of WBCs, as well as RBC counts and parameters. These resulted in some degree of physiological and functional anemia, likely through oxidative stress. However, most of these disturbances were normalized after three weeks of treatment. More importantly, the route of administration has a major effect on the induction of hematological disturbances, and consequently, it should be considered when ENPs are applied as drug delivery systems.

## Figures and Tables

**Table 1.** The effect of oral administration and intraperitoneally (i.p.) injection of ENPs on white blood cells (WBCs) and deferential counts in rats. PLT, platelet.

Parameter	PLT (10^3^/cc)		WBCs (10^3^/cc)		Lymph (%)		Mono (%)		Gran (%)	

Group	Oral	IP	Oral	IP	Oral	IP	Oral	IP	Oral	IP
Control	341 ± 28^aA^	389 ± 16^abA^	5.8 ± 0.55^bA^	5.77 ± 0.26^aA^	85.8 ± 1.66^aA^	85.3 ± 1.81^aA^	6.73 ± 0.32^aA^	6.40 ± 0.41^aA^	8.10 ± 0.95^aA^	8.30 ± 1.5^aA^
4 h	408 ± 19^aA^	222 ± 7.4^bB^	4.8 ± 1.12^bA^	5.76 ± 0.45^aA^	84.2 ± 4.1^aA^	76.46 ± 6.62^aA^	6.73 ± 0.55^aA^	6.37 ± 0.70^aA^	9.06 ± 1.19^aA^	17.16 ± 2.63^aB^
48 h	492 ± 39^aA^	437 ± 67^aA^	7.27 ± 1.11^aA^	8.76 ± 0.64^aB^	78.10 ± 3.0^aA^	82.26 ± 4.0^aA^	6.07 ± 0.81^aA^	6.30 ± 1.05^aA^	15.83 ± 1.19^aA^	11.43 ± 3.04^aB^
One week	473 ± 42^aA^	451 ± 56^abA^	6.80 ± 0.31^aA^	7.46 ± 0.75^aB^	80.40 ± 2.95^aA^	82.66 ± 1.61^aA^	6.57 ± 0.27^aA^	7.06 ± 0.36^aA^	13.03 ± 2.69^aA^	10.27 ± 1.97^aB^
Three weeks	457 ± 55^aA^	455 ± 56^aA^	5.17 ± 0.51^bA^	6.63 ± 1.66^aB^	81.83 ± 2.57^aA^	83.20 ± 2.65^aA^	7.37 ± 0.53^aA^	6.97 ± 1.07^aA^	10.80 ± 0.15^aA^	9.83 ± 1.60^aA^

All data are expressed as the mean ± standard error; means with different superscript letters (a, b) are significantly different (*p* ≤ 0.05); lowercase letters are for groups within the same column, while uppercase letters (A, B) to compare oral treatment with IP treatment within the same row.

**Table 2.** The effect of oral administration and i.p. injection of ENPs on red blood cells (RBCs) and hematological parameters in rats. Hct, hematocrite; MCV, mean corpuscular volume; MCH, mean corpuscular hemoglobin; MCHC, mean corpuscular hemoglobin concentration.

Parameter	RBCs (10^6)^		Hb (g/dL)		HCt (%)		MCV (fL)		MCH (pg)		MCHC (g/dL)	
	
group	Oral	IP	Oral	IP	Oral	IP	Oral	IP	Oral	IP	Oral	IP
Control	5.42 ± 0.46^aA^	5.77 ± 0.55^aA^	14.60 ± 0.58^aA^	13.01 ± 1.58^aA^	42.4 ± 1.3^aA^	36.7 ± 3.13^aA^	47.73 ± 3.13^bA^	51.3 ± 1.52^bA^	29.6 ± 1.17^aA^	28.3 ± 3.98^aA^	34.4 ± 1.96^aA^	31.7 ± 4.40^bA^
4 h	5.41 ± 0.26^aA^	4.43 ± 0.90^bA^	14.93 ± 0.88^aA^	13.73 ± 1.45^aA^	42.37 ± 1.25^aA^	30.4 ± 4.43^bA^	45.33 ± 1.88^cA^	51.0 ± 3.85^bA^	27.4 ± 0.56^aA^	31.60 ± 3.30^aA^	33.4 ± 1.56^aA^	32.3 ± 1.69^abA^
48 h	4.50 ± 0.26^bA^	4.95 ± 0.33^aA^	13.46 ± 1.21^abA^	13.8 ± 0.87^aA^	34.66 ± 1.23^aA^	32..4 ± 3.83^bA^	57.66 ± 4.84^bA^	62.33 ± 6.99^bA^	31.03 ± 1.84^aA^	34.83 ± 2.45^aA^	36.13 ± 3.08^aA^	41.2 ± 3.71^abA^
One week	6.18 ± 0.72^aA^	5.48 ± 0.26^aA^	10.80 ± 1.50^bA^	9.47 ± 0.41^aA^	41.20 ± 2.37^aA^	35.33 ± 1.56^aA^	133.6 ± 2.02^aA^	129.0 ± 1.73^aA^	24.60 ± 1.10^aA^	24.53 ± 1.41^aA^	51.9 ± 2.36^aA^	35.5 ± 1.27^aA^
Three weeks	5.75 ± 0.88^aA^	6.41 ± 0.49^aA^	11.86 ± 0.87^bA^	11.40 ± 1.51^aA^	38.13 ± 1.98^aA^	35.2 ± 2.11^aA^	56.33 ± 1.33^bA^	57.3 ± 1.53^bA^	18.9 ± 1.14^bA^	28.46 ± 1.57^aA^	33.7 ± 1.32^aA^	32.3 ± 1.95^bA^

All data are expressed as the mean ± standard error; means with different superscript letters are significantly different (*p* ≤ 0.05); lowercase letters (a, b) are for groups within the same column, while uppercase letter (A) to compare oral treatment with IP treatment within the same row.

**Table 3.** The effect of oral administration and i.p. injection of ENPs on Hb derivatives (expressed as percent of total hemoglobin) in rats.

parameter	Oxy-Hb		MetHb		S-Hb		Co-Hb	

group	Oral	IP	Oral	IP	Oral	IP	Oral	IP
Control	95.94 ± 0.87^aA^	96.24 ± 1.05^aA^	3.56 ± 0.74^aA^	3.22 ± 0.74^aA^	0.120 ± 0.01^aA^	0.118 ± 0.03^aA^	0.36 ± 0.18^aA^	0.432 ± 0.14^aA^
4 h	95.83 ± 1.75^aA^	95.17 ± 0.74^aA^	3.47 ± 0.80^aA^	3.43 ± 0.79^aA^	0.114 ± 0.03^aA^	0.115 ± 0.04^aA^	0.624 ± 0.20^bA^	0.688 ± 0.19^bA^
48 h	95.39 ± 1.13^aA^	95.65 ± 1.11^aA^	3.83 ± 0.67^aA^	3.64 ± 0.69^aA^	0.099 ± 0.02^aA^	0.120 ± 0.03^aA^	0.678 ± 0.19^bA^	0.639 ± 0.21^bA^
One week	95.72 ± 1.75^aA^	95.42 ± 2.78^aA^	3.88 ± 0.80^aA^	3.82 ± 0.83^aA^	0.117 ± 0.04^aA^	0.122 ± 0.04^aA^	0.682 ± 0.22^bA^	0.709 ± 0.23^bA^
Three weeks	95.49 ± 1.32^aA^	95.05 ± 1.27^aA^	3.99 ± 0.83^aA^	4.41 ± 0.79^bA^	0.156 ± 0.02^aA^	0.128 ± 0.01^aA^	0.400 ± 0.11^aA^	0.404 ± 0.12^aA^

All data are expressed as the mean ± standard error; means with different superscript letters are significantly different (*p* ≤ 0.05); lowercase letters (a, b) are for groups within the same column, while uppercase letter (A) to compare oral treatment with IP treatment within the same row.
